# Susceptibilities of medaka (*Oryzias latipes*) cell lines to a betanodavirus

**DOI:** 10.1186/1743-422X-7-150

**Published:** 2010-07-12

**Authors:** Kei Adachi, Kosuke Sumiyoshi, Ryo Ariyasu, Kasumi Yamashita, Kosuke Zenke, Yasushi Okinaka

**Affiliations:** 1 Graduate School of Biosphere Science, Hiroshima University, Higashi-hiroshima 739-8528, Japan

## Abstract

**Background:**

Betanodaviruses, members of the family *Nodaviridae*, have bipartite, positive-sense RNA genomes and are the causal agents of viral nervous necrosis in many marine fish species. Recently, the viruses were shown to infect a few freshwater fish species including a model fish medaka (*Oryzias latipes*). Although virological study using cultured medaka cells would provide a lot of insight into virus-fish interactions in molecular aspects, no such cells have yet been tested for virus susceptibility.

**Results:**

We tested ten medaka cell lines for susceptibilities to redspotted grouper nervous necrosis virus (RGNNV). Although the viral coat protein was detected in all the cell lines inoculated, the levels of cytopathic effect development and viral propagation were quite different among the cell lines. Those levels were especially high in OLHNI-1 and OLHNI-2 cells, but were extremely low in OLME-104 cells. Some cell lines entered into antiviral state after RGNNV infections probably because of inducing an antiviral system. This is the first report to examine the susceptibilities of cultured medaka cells against a virus.

**Conclusion:**

OLHNI-1 and OLHNI-2 cells are candidates of new standard cells for betanodavirus study because of their high susceptibilities to the virus and their several advantages as model fish cells.

## Background

Betanodaviruses, members of the family *Nodaviridae*, are small non-enveloped viruses with a genome composed of a bipartite single-stranded, positive-sense RNA [1,2]. The larger genomic segment, RNA1 (3.1 kb), encodes the RNA-dependent RNA polymerase and the smaller genomic segment, RNA2 (1.4 kb), encodes the coat protein (CP) [2]. During viral RNA replication, a subgenomic RNA3 is produced, which encodes the RNA interference inhibitor protein B2 [3-5]. Betanodaviruses are classified basically into four genotypes based on the phylogenetic analysis of their genomic RNA2 sequences [6-8]. These genotypes are striped jack nervous necrosis virus (SJNNV), barfin flounder nervous necrosis virus (BFNNV), tiger puffer nervous necrosis virus (TPNNV) and redspotted grouper nervous necrosis virus (RGNNV). Recently, a betanodavirus isolate from turbot (*Scophthalmus maximus*) was suggested to belong to a fifth genotype [9].

Betanodaviruses are the causative agents of a highly destructive disease of marine fish designated viral nervous necrosis. The viruses have been isolated from a large number of marine fish species [10,11]. Betanodaviruses propagate in various established cell lines derived from not only fish [2,12] but also mammals [13]. Recently, it was revealed that larvae of freshwater fish guppy (*Poicelia reticulata*) [14] and tilapia (*Oreochromis niloticus*) [15] were affected naturally by RGNNV. Some freshwater fish including medaka (*Oryzias latipes*) [16,17] and zebrafish (*Danio rerio*) [18] are lethally susceptible to betanodaviruses under experimental conditions. Medaka has several experimental advantages as a model fish compared to other fish and higher vertebrates. For example, medaka is small, cost-effective, easy to breed in large numbers, and has a short life cycle. Furthermore, whole medaka genomic sequences are available and many experimental techniques for gene function analysis can be applied to medaka [19,20]. However, one obstacle to study betanodavirus-medaka interactions in molecular aspects is the lack of cultured medaka cells which are susceptible to a betanodavirus. Therefore, in this study, we examined the susceptibilities of ten medaka cell lines derived from different strains and organs to RGNNV.

## Results

### Virus infection and cytopathic effect (CPE) development

**Figure 1:**
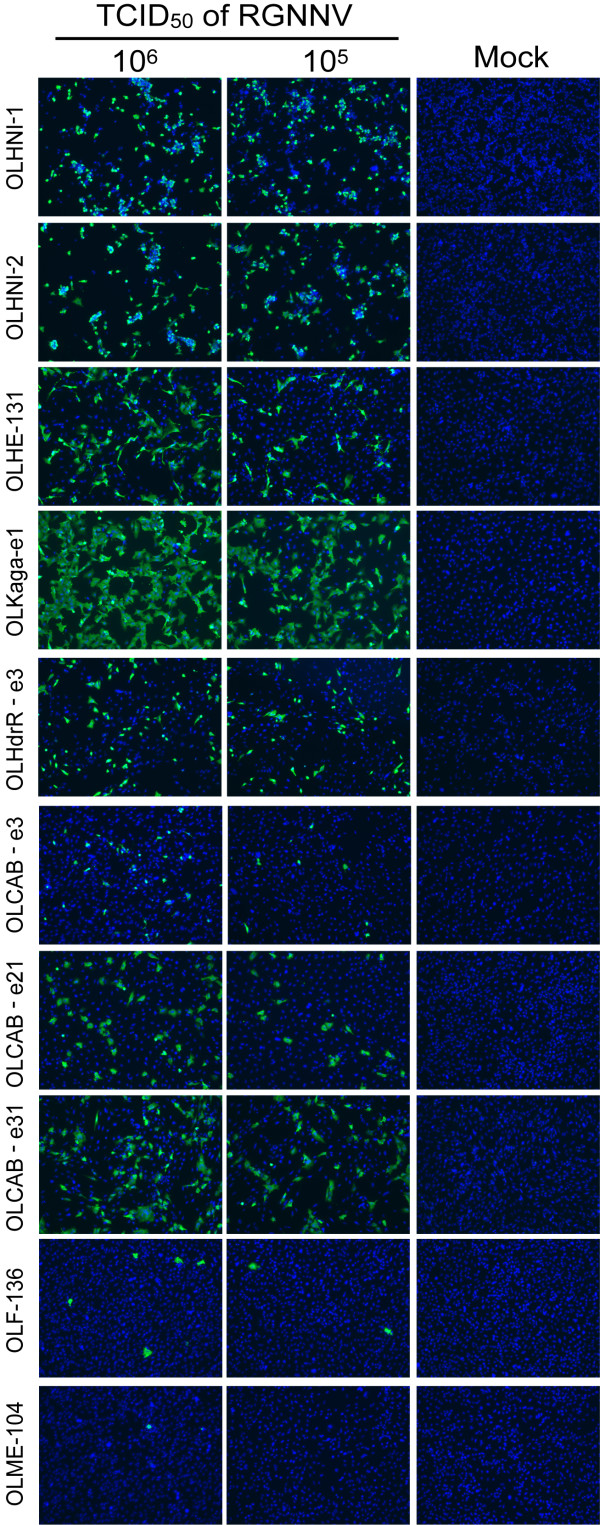
**Infectivity of RGNNV in various medaka cell lines**. Each cell line (1.0-1.5 × 10^5 ^cells) was inoculated with 10^5 ^or 10^6 ^TCID_50 _of RGNNV and incubated at 30°C. Viral coat protein in infected cells was detected by indirect immunofluorescence assay at 1 dpi. Cell nucleus was stained with 4', 6-diamino-2-phenylindole (DAPI). Data represents the merged image of Alexa488-fluorencence and DAPI staining.

**Figure 2:**
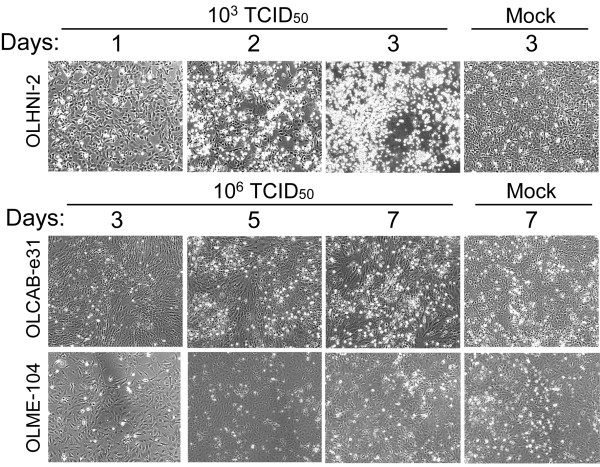
**CPE development in RGNNV-infected medaka cells**. The cells (1.0-1.5 × 10^5^) were inoculated with RGNNV of the indicated titers and cultured at 30°C. Cell morphology of the RGNNV-inoculated or mock-inoculated cells was observed at 1-3 dpi for OLHNI-2 cells and at 3-7 dpi for OLCAB-e31 and OLME-104 cells.

We firstly examined the infectivity of RGNNV against the medaka cell lines (Table 1) by detecting CP-expressing cells at 1 day post-inoculation (dpi). When the cells were inoculated with RGNNV having the 50% tissue culture infectious dose (TCID_50_) of 10^6^, most of the inoculated cells expressed the CP in OLHNI-1, OLHNI-2, and OLKaga-e1 cells (Figure 1). In contrast, quite a small number of cells expressed the CP in OLF-136 and OLME-104 cells (Figure 1). The typical CPE, represented as rounded cells which were finally detached from the dish, was detected only in OLHNI-1 and OLHNI-2 cells at 1 dpi of 10^5 ^or 10^6 ^TCID_50 _of RGNNV (data not shown). To examine further whether the eight cell lines other than OLHNI-1 and OLHNI-2 cells exhibit CPE by RGNNV-inoculations, inoculated cells were incubated for up to 7 days and observed under a microscope (Figure 2). OLHNI-2 cells showed the apparent CPE at 2 dpi when the cells were exposed to 10^3 ^TCID_50 _of RGNNV and almost detached from the dish at 3 dpi (Figure 2). OLHNI-1, OLHE-131, OLKaga-e1, and OLHdrR-e3 cells also showed apparent CPE in 4-5 days after inoculated with 10^3 ^TCID_50 _of virus (data not shown). In contrast, no apparent CPE was observed in OLCAB-e31, OLME-104, OLCAB-e21, or OLF-136 cells (Figure 2, data not shown for the latter two) within 7 days even though the cells were exposed to 10^6 ^TCID_50 _of virus. These results indicate that OLHNI-1 and OLHNI-2 cells are highly susceptible to RGNNV compared with the other cell lines. Furthermore, RGNNV production and/or spread seem to be restricted in some of the medaka cell lines though the virus can multiply in all of the cell lines to a varying degree.

**Table 1:** Medaka cell lines used in this study

Cell line	Species	Strain	Tissue derived
OLHNI-1	*Oryzias latipes*	HNI	Caudal fin
OLHNI-2	*O. latipes*	HNI	Caudal fin
OLHE-131	*O. latipes*	H04C	Liver
OLKaga-e1	*O. latipes*	Kaga	Embryo
OLHdrR-e3	*O. latipes*	Hd-rR	Embryo
OLCAB-e3	*O. latipes*	Cab	Embryo
OLCAB-e21	*O. latipes*	Cab	Embryo
OLCAB-e31	*O. latipes*	Cab	Embryo
OLF-136	*O. latipes*	Unknown	Fin
OLME-104	*O. latipes*	HB32C	Melanoma

### Virus spread

**Figure 3:**
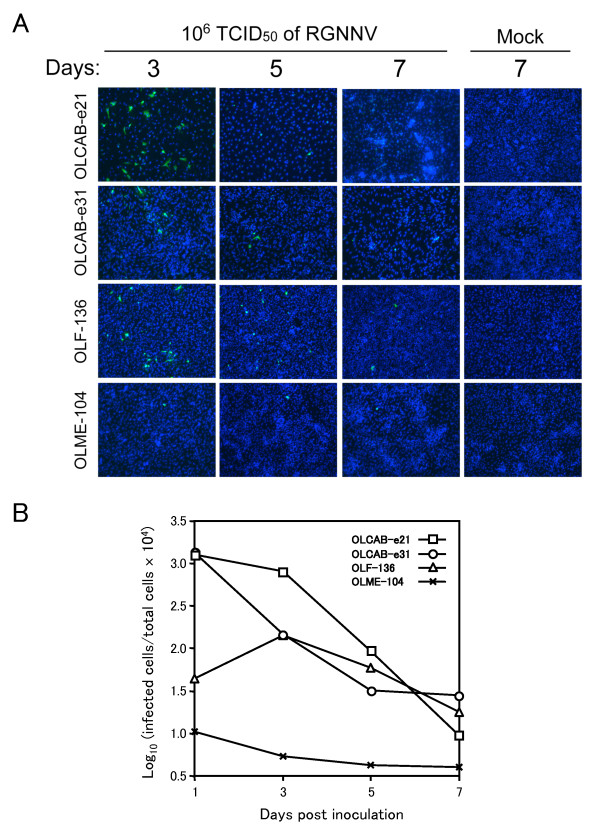
**Restriction of RGNNV spread among medaka cells**. (A) The cells (1.0-1.5 × 10^5^) were inoculated with 10^6 ^TCID_50 _of RGNNV and cultured at 30°C. The CP and cell nucleus in the infected cells were detected by indirect immunofluorescence assay and DAPI staining, respectively, at the indicated period. Data represents the merged image of Alexa488-fluorescence and DAPI staining. (B) Rates for the infected cells against the total cells represented in Figures 1 and 3A were calculated and shown periodically.

To examine whether RGNNV spread occur in the medaka cell lines which lacked clear appearance of CPE (Figure 2), we examined CP-expressing cells in those cell lines inoculated with RGNNV. In OLCAB-e21 and OLCAB-e31 cells, the numbers of CP-expressing cells were decreased dramatically with time (Figure 3) compared with those at 1 dpi (Figure 1). In OLF-136 cells, the number of CP-expressing cells was increased transiently at 3 dpi (Figure 3) compared to that at 1 dpi (Figure 1) but then decreased gradually. No virus spread was observed in OLME-104 cells throughout the experimental period (Figures 1 and 3). These results indicate that the viral spread was tightly limited in the four cell lines, which resulted in the defect of apparent CPE development as shown in Figure 2.

### Production of progeny virus

In DBT cells which are derived from murine astrocytoma, RGNNV propagated efficiently without substantial increases in the number of virus-infected cells and CPE-exhibiting cells [13]. These data suggest that a great amount of RGNNV production is not necessarily concerned with the number of virus-infected cells and appearance of CPE. To quantify progeny virus from inoculated medaka cells, we calculated the viral titers in the culture supernatant by the TCID_50 _method (Table 2). The viral titers of the culture supernatant from infected OLHNI-1 and OLHNI-2 cells increased sequentially and reached more than 10^9 ^TCID_50_/ml at 5 dpi. Similarly, the maximum viral titers were approximately: 10^8 ^TCID_50_/ml in OLHE-131, OLKaga-e1, and OLHdrR-e3 cells; 10^7 ^TCID_50_/ml in OLCAB-e3, OLCAB-e31, and OLF-136 cells; 10^6 ^TCID_50_/ml in OLCAB-e21 cells. The viral titers of the supernatant from inoculated OLME-104 cells did not increase during the whole experimental period, suggesting that RGNNV was unable to propagate in this cell. Interestingly, in E-11 cells, the maximum viral titer was 10^8.1 ^TCID_50_/ml, which was approximately 10-fold lower than those of OLHNI-1 and OLHNI-2 cells. These results indicated that RGNNV multiplies in various medaka cell lines, especially in OLHNI-1 and OLHNI-2 cells, but hardly in OLME-104 cells. Furthermore, OLHNI-1 and OLHNI-2 cells are candidates of new standard cells for betanodavirus study because of their high susceptibilities to the virus and their several advantages as model fish cells.

**Table 2:** Betanodavirus propagation in the medaka cell lines

Cell line	Inoculum ^a ^(TCID_50_)	Viral titer (TCID_50_/ml) ^b^			
		
		Day 1	Day 3	Day 5	Day 7
OLHNI-1	10^3^	4.4 × 10^4 ^± 1.2 × 10^4^	4.9 × 10^8 ^± 7.0 × 10^7^	1.8 × 10^9 ^± 7.5 × 10^8^	NT
OLHNI-2	10^3^	4.0 × 10^4 ^± 0	4.0 × 10^8 ^± 1.7 × 10^8^	1.4 × 10^9 ^± 4.0 × 10^8^	NT
OLHE-131	10^3^	3.3 × 10^3 ^± 7.5 × 10^2^	4.4 × 10^7 ^± 1.2 × 10^7^	2.6 × 10^8 ^± 6.0 × 10^7^	NT
OLKaga-e1	10^3^	4.0 × 10^3 ^± 0	2.9 × 10^8 ^± 3.5 × 10^7^	3.2 × 10^8 ^± 8.5 × 10^7^	NT
OLHdrR-e3	10^3^	4.8 × 10^3 ^± 8.0 × 10^2^	1.9 × 10^8 ^± 1.0 × 10^7^	2.9 × 10^8 ^± 3.5 × 10^7^	NT
OLCAB-e3	10^6^	2.9 × 10^5 ^± 3.5 × 10^4^	2.1 × 10^7 ^± 4.0 × 10^6^	2.5 × 10^7 ^± 1.5 × 10^7^	3.6 × 10^7 ^± 4.0 × 10^6^
OLCAB-e21	10^6^	5.1 × 10^4 ^± 1.9 × 10^4^	4.1 × 10^6 ^± 1.6 × 10^6^	3.7 × 10^6 ^± 1.9 × 10^6^	1.9 × 10^6 ^± 1.3 × 10^6^
OLCAB-e31	10^6^	3.6 × 10^5 ^± 4.0 × 10^4^	5.6 × 10^6 ^± 0	5.6 × 10^6 ^± 0	7.8 × 10^6 ^± 2.2 × 10^6^
OLF-136	10^6^	1.1 × 10^5 ^± 6.9 × 10^4^	4.8 × 10^6 ^± 8.0 × 10^5^	7.8 × 10^6 ^± 2.2 × 10^6^	2.1 × 10^7 ^± 1.1 × 10^7^
OLME-104	10^6^	4.8 × 10^4 ^± 8.0 × 10^3^	4.8 × 10^4 ^± 8.0 × 10^3^	3.6 × 10^4 ^± 4.0 × 10^3^	3.6 × 10^4 ^± 4.0 × 10^3^
E-11	10^3^	2.7 × 10^4 ^± 1.3 × 10^4^	1.4 × 10^8 ^± 1.3 × 10^8^	1.4 × 10^8 ^± 4.0 × 10^7^	NT

a. Medaka cells and E-11 cells (1.0-1.5 × 10^5^) were inoculated with RGNNV of the indicated titers.

b. Viral titers in the culture supernatant were determined by the TCID_50 _assay using E-11 cells at 30°C.

Each data represents the mean and standard deviation from two independent experiments.

NT, Not tested.

## Discussion

We have demonstrated the susceptibilities of established medaka cells to the betanodavirus (RGNNV), the levels of which varied irrespective of the originated tissues. Medaka cell lines could be classified into three categories in terms of the infectivity and/or the productivity of RGNNV in the cells as follows: (1) cells are efficiently infected by virus, and give CPE and a high titer of progeny virus as is the cases for OLHNI-1, OLHNI-2, OLHE-131, OLKaga-e1, and OLHdrR-e3 cells, (2) cells are infected by virus though CPE and viral spread are tightly limited, which resulted in production of a low amount of progeny virus as is the cases for OLCAB-e3, OLCAB-e21, OLCAB-e31 and OLF-136 cells, (3) cells are hardly infected by virus as is the cases for OLME-104 cells. There would be two possible processes which determined the levels of the susceptibilities to RGNNV. One is the presence or absence of cellular factors required for RGNNV infection, such as cell-specific receptors. The other is the presence or absence of cellular factors which repress RGNNV infection. With regard to the former possibility, fibronectin 2 of zebrafish (*Danio rerio*) is the only cellular factor which has so far been identified for fish viruses. Zebrafish fibronectin 2 mediates infectious hematopoietic necrosis virus attachment and cell entry [21]. Cell-surface sialic acid is involved in binding of RGNNV to SSN-1 cells and other cellular molecules are required along with sialic acid for RGNNV penetration into some human cell lines [22,23]. However, such a cellular molecule essential for betanodavirus infection has not yet been identified. OLME-104 cells were severely less susceptible to RGNNV (Figure 1), suggesting the lacks of specific cellular factors for RGNNV. Meanwhile, OLHNI-1 and OLHNI-2 cells were highly susceptible to RGNNV (Figure 1), suggesting that these cell lines possess a positive cellular factor for betanodavirus infection. With respect to the latter possible mechanism, the culture supernatant of OLME-104 cells included an antiviral substance that protected some of the medaka cell lines from RGNNV-infection (authors' unpublished data). These results indicate that OLME-104 cells produce an interferon-like signal molecule irrespective of viral infection, which brings themselves into antiviral state. Furthermore, these data also indicate that some medaka cells used in this study are sensitive to such a defense signal molecule.

The OLCAB-e21, OLCAB-e31, and OLF-136 cells infected by RGNNV produced sufficient amounts of infectious virus particles in the culture supernatant (Table 2) though the levels of CPE and viral spread were tightly limited (Figures 2 and 3). These characteristics suggest that the cells entered into antiviral states after viral infections. Similar to mammalian Mx proteins, fish Mx proteins also possess type I interferon (IFN)-inducible antiviral activity in vitro and in vivo [24-27]. Grouper (*Epinephelus coioides*) Mx proteins inhibited the propagation of RGNNV in the grouper brain cells [28]. In addition, the BB cell line derived from the brain of barramundi (*Lates calcarifer*) was infected persistently with RGNNV and this viral persistence in BB cells was well correlated with the expression of Mx gene [29,30]. Thus, an IFN-like substance might be produced in the culture supernatant of the RGNNV-infected OLCAB-e21, OLCAB-e31, and OLF-136 cells, which induces the cells into antiviral states. However, Mx gene expression was detected by RT-PCR in OLCAB-e31 cells inoculated with RGNNV, not in inoculated OLCAB-e21 or OLF-136 cells (authors' unpublished data). These results suggest that a defense machinery other than the IFN system works in OLCAB-e21 and OLF-136 cells. Taken together, a few kinds of defense systems could function to protect the medaka cells from RGNNV infection.

E-11 cells [12] cloned from SSN-1 cells are infected latently with snakehead retrovirus (SnRV) [31]. SnRV regulates positively [32] or negatively [33] the infections of fish cells with betanodaviruses. In our experiments, SnRV was detected by RT-PCR in all of the medaka cells inoculated with RGNNV prepared from infected E-11 cells. However, there was no correlation between susceptibilities of medaka cells to RGNNV and the levels of RT-PCR signals for SnRV (authors' unpublished data).

## Conclusions

In this report, we examined the susceptibility of various medaka cell lines to RGNNV, and found that RGNNV can infect and propagate in many kinds of established medaka cells. Studies on host-betanodavirus interactions using these medaka cell lines would lead to the identification of host factors essential for betanodavirus infections. Especially, OLHNI-1 and OLHNI-2 cells would be suitable for such studies in molecular aspects.

## Methods

### Cells and viruses

The three medaka cell lines, OLHE-131, OLF-136, and OLME-104 (Table 1), were purchased from RIKEN BRC Cell Bank (Tsukuba, Japan). The other seven medaka cell lines (Table 1) [34] were provided from H. Mitani. All the medaka cells were cultured at 30°C in Leibovitz's L-15 medium (L-15) (Invitrogen, Carlsbad, CA, USA) containing 15% fetal bovine serum (FBS). E-11 cells [12] were cultured in L-15 medium supplemented with 5% FBS. The betanodavirus used in this study was RGNNV (SGWak97 strain) [35]. Virus was prepared from the inoculated E-11 cells when more than 90% of the inoculated cells showed CPE. Viral titers were determined based on TCID_50 _[36] using E-11 cells.

### Viral inoculation and multiplication assay

Medaka cells were seeded in 24-well plates and were inoculated with RGNNV at 30°C for 1 h. For each cell line, 1.0-1.5 × 10^5 ^cells were inoculated with 10^3^, 10^5^, or 10^6 ^TCID_50 _of virus. The cells were washed to remove unbound viral particles and were further cultured at the same temperature. The culture supernatant was recovered periodically and its viral titer was determined by the TCID_50 _assay as described above.

### Immunofluorescence microscopy

Indirect immunofluorescence assay was performed using inoculated medaka cells as described previously [22]. Briefly, cells were fixed with 4% paraformaldehyde and permeabilized by treatment with 0.1% NP-40 in PBS. The cells then were treated with a 1:1000 dilution of anti-RGNNV CP antiserum, followed by the treatment with a 1:2000 dilution of Alexa Fluor 488 goat anti-rabbit IgG (Invitrogen), and were observed under the fluorescence microscope (ORCA-1394 and AQUA-Lite version 1.10 systems; Hamamatsu photonics K. K., Hamamatsu, Japan).

## Competing interests

The authors declare that they have no competing interests.

## Authors' contributions

KA participated in design and interpretation of the experiments, performed the research, and wrote the manuscript. KS and KZ carried out a part of the virological experiments. RA and KY participated in cell culture and preparation of the virus sample. YO conceived of the study, and was involved in the design and cordination. All authors approved the final version of the manuscript.
